# Anti-inflammatory effects of extracellular vesicles from *Morchella* on LPS-stimulated RAW264.7 cells via the ROS-mediated p38 MAPK signaling pathway

**DOI:** 10.1007/s11010-022-04508-y

**Published:** 2022-07-07

**Authors:** Qi Chen, Chengchuan Che, Shanshan Yang, Pingping Ding, Meiru Si, Ge Yang

**Affiliations:** College of Life Sciences, Qufu Normal University, Qufu, Shandong 273165 People’s Republic of China

**Keywords:** *Morchella*, Extracellular vesicles, Anti-inflammatory, p38 MAPK pathway

## Abstract

*Morchella* is a kind of important edible and medicinal fungi, which is rich in polysaccharides, enzymes, fatty acids, amino acids and other active components. Extracellular vesicles (EVs) have a typical membrane structure, and the vesicles contain some specific lipids, miRNAs and proteins, and their can deliver the contents to different cells to change their functions. The present study investigated whether *Morchella* produce extracellular vesicles and its anti-inflammatory effect on lipopolysaccharide (LPS)-induced RAW246.7 macrophages. The experimental results showed that *Morchella* produced extracellular vesicles and significantly reduced the production of nitric oxide (NO) and reactive oxygen species (ROS) in a model of LPS-induced inflammation. In addition, the expression of inflammatory factor-related genes such as inducible nitric oxide synthase (iNOS), tumor necrosis factor-α (TNF-α), interleukin-6 (IL-6) and cyclooxygenase-2 (COX-2) showed dose-dependent inhibition. *Morchella* extracellular vesicles also can inhibit the inflammatory response induced by LPS by inhibiting the production of ROS and reducing the phosphorylation levels of the p38 MAPK signaling pathway. These results indicate that the *Morchella* extracellular vesicles can be used as a potential anti-inflammatory substance in the treatment of inflammatory diseases.

**Supplementary Information:**

The online version contains supplementary material available at 10.1007/s11010-022-04508-y.

## Introduction

Extracellular vesicles (EVs) have been shown to be released by different types of cells to communicate with each other, such as mammals, microbes and parasites. These vesicles mediate communication between cells by delivering their contents such as proteins, microRNAs and lipids [1]. Studies have proven that fungal EV and mammalian EV show a strong similarity in molecular content [2]. The characterization of EVs has been reported in both pathogenic and non-pathogenic fungi [3–7]. There are increasing reports of beneficial roles of EVs secreted by probiotic bacteria. For example, *Lactobacillus rhamnosus* GG-derived EVs have been associated with the apoptosis of hepG2 cancer cells [8], *Lactobacillus plantarum* WCFS1-derived EVs modulated the response of human cells to vancomycin-resistant enterococci [9] and Extracellular Vesicles Produced by the Probiotic *Propionibacterium freudenreichii* CIRM-BIA 129 Mitigate Inflammation by Modulating the NF-κB Pathway [10].

*Morchella* belongs to the species of *Morchella*, genus of *Morchellaceae*, family of *Oncobacteria*, subdivision of *Ascomycetes* [11]. There are a wide variety of fungi in the genus *Morchella*, which is recognized as one of the world's most precious and rare edible mushrooms. *Morchella* is a kind of important edible and medicinal fungi, which is rich in polysaccharides, enzymes, fatty acids, amino acids and other active components [12–17]. The indigestion, excessive phlegm and shortness of breath were treated by *Morchella* in ancient Chinese. All varieties of *Morchella* are rare edible and medicinal fungi with delicious taste, rich nutrients and high medicinal value [18–20]. Some studies suggest that the extracted *Morchella* conica polysaccharides (MCP) from *Morchella* may act as a potent immunomodulatory agent to modulate NO production in macrophages and it also promotes the proliferation of splenocytes [21]. An article reported that acetylation of polysaccharides from *Morchella* was able to greatly enhance the anti-inflammatory activity of macrophages [22].

Inflammation is an important physiological defense response of our body in response to external stimuli [23]. The body's defense response is inseparable from the activation of macrophages [24]. It is complex and difficult for inflammatory diseases to cure so that a good model of inflammation is important for subsequent experiments. Mouse monocyte macrophage leukemia cells (RAW264.7 cells), a typical macrophage, are widely used for in vitro inflammation modeling and can be activated by various stimuli such as LPS and inflammatory factors [25]. Lipopolysaccharide (LPS) is an endotoxin and a component of the cell wall of Gram-negative bacteria. LPS is able to activate several inflammatory signaling pathways, thus regulating the expression and release of inflammatory mediators and inflammation-related enzymes, and is often used as an inducer to induce inflammation in macrophages [26–30]. When LPS is used to model inflammation and it is recognized and bound by the relevant receptors on the surface of macrophages, which triggers a series of physiological and biochemical reactions in the cells, leading to a large release of some inflammatory factors (TNF-α, IL-6, iNOS, etc.) and causing local inflammation [31, 32]. NO acts as a specific intercellular signaling molecule in the organism and can elicit specific intracellular responses [33]. When inflammation occurs in the body increased expression of iNOS, it can catalyze the production of NO, which in turn leads to further development of inflammation [34]. TNF-α is a cytokine involved in systemic inflammation and is mainly secreted by macrophages [35]. IL-6 is a promoter of inflammatory response can stimulate an increased inflammatory response, which makes it an important inflammatory assay marker [36, 37]. Cyclooxygenase-2 (COX-2) is induced by cytokines, endotoxins and growth factors in macrophages, and it can changes arachidonic acid to prostanoids which can cause pain and swelling at the site of inflammation [38, 39]. ROS are key players in immune cell signaling [40]. LPS can induce an early ROS production, which can as the original signal to enhance the MAPK activation [41–43].

LPS stimulates macrophages through activation of mitogen-activated protein kinase (MAPK) signaling pathway, thereby causing an inflammatory response [29]. MAPK signaling pathway plays a key role in normal physiological functions of cells such as the regulation of cell survival, cell proliferation and cell differentiation. In addition, it regulates the expression of nitrogen oxide (NO), inducible nitric oxide synthase (iNOS), cyclooxygenase-2 (COX-2), IL-6 and TNF-α, which are genes associated with inflammation [44]. Studies have shown that MAPK signaling pathway includes three branching routes: extracellular signal-regulated kinase (ERK), c-Jun N-terminal kinase stress-activated protein kinase (JNK), p38 [45]. The most important member of the MAPK family in regulating inflammatory response is p38, which can detect quantities of extracellular signaling stimuli such as LPS [46]. Reactive oxygen species (ROS) is a major participant in oxidative stress and involved in pathological processes including cellular inflammation. p38 MAPK signaling pathway can be activated by adverse stimuli, such as ROS, and mediate inflammatory responses [47].

Here, we examined the anti-inflammatory effects of *Morchella* EVs on LPS-induced inflammation in RAW264.7 cells. In addition, we evaluated whether *Morchella* EVs regulates p38 MAPK pathways and whether it is also related to reactive oxygen species to prove the inhibitory mechanism underlying their anti-inflammatory effect.

## Materials and methods

### Chemicals and reagents

LPS (lipopolysaccharide) were purchased from Sigma Aldrich Inc (St. Louis, United States), 4′,6-diamidino-2-phenylindole (DAPI) and DIO were obtained from Coollaber Technology (Beijing, China). Dulbecco's modified eagle medium (DMEM) and Fetal Bovine Serum was purchased from Biological Industries (Kibbutz Beit Haemek, Israel). 4% Paraformaldehyde Fix Solution, reactive oxygen species (ROS) assay kit, NO assay kit and BCA assay kit were obtained from Beyotime Biotech-nology Co., Ltd. (Shanghai, China). Quantitative real time polymerase chain reaction (RT-qPCR) reagents were from Vazyme Biotech Co., Ltd. (Beijng, China). DMSO and MTT were from Sangon BiotechCo., Ltd. (Shanghai, China). Antibodies for p38 MAPK, MK2 and β-actin are mouse-derived monoclonal antibodies purchased from Proteintech Group (Wuhan, China). Antibody for CD81, CD63 and Phospho-p38 MAPK (p-p38 MAPK) are rabbit-derived polyclonal antibody purchased from the affinity biosciences LTD (Cincinnati, USA). Secondary antibodies are goat anti-rabbit HRP (Affinity Biosciences, Cincinnati, USA) and goat anti-mouse HRP (Proteintech Group, Wuhan, China), respectively.

### Activation and culture of strains

*Morchella* is from the existing *Morchella Sextelata* (MG431334.1) in this experiment. Firstly, the strains were removed and transferred to PDA solid medium (fresh potato 20%, glucose 2%, AGAR 2%) for activation, and cultured in an incubator at 25 ℃ for 2 days. When spores grow mycelium, the mycelium is inoculated from the old PDA medium to the new PDA medium, so that it continues to grow. The above steps should be repeated until a single mycelium is obtained. Mycelia from solid PDA medium were selected, transferred to PDA liquid medium, and placed in shaker culture at 18 ℃, 180 r/min.

### Isolation and characterization of *Morchella* EVs

The separation method of *Morchella* EVs was use for reference Vallejo et al. [7] and slightly modified. First, *Morchella* was cultured in PDA liquid medium for 72 h. At the end of the culture, the bacterial solution was separated into Amicon Ultra-15 ultrafiltration tube (100 kDA, Merck Millipore, Billerica, MA, USA) and centrifuged at 3000 × *g* for 30 min at 4 ℃. Then, the supernatant obtained was centrifuged at 12,000 × *g* for 15 min at 4 ℃. And supernatant was ultrancentrifuged (Beckman Coulter, Inc., XPN-90, California, USA), at 150,000 × *g* for 1 h at 4 ℃. *Morchella* EVs dissolved in suitable amount of sterile nuclease-free PBS. Size exclusion chromatography was used for the purification of extracellular vesicles. The EVs collected after ultracentrifugation were added to IZON qEVs (qEVs single, Izon Science, Oxford, UK) and eluted using PBS (pH: 7.4, 0.22 µm filter). Fractions 7–9 were collected according to the manufacturer's instructions. The fractions were further concentrated by centrifugation at 3000 × *g* for 30 min at 4 °C using Amicon Ultra-4 ultrafiltration tube (10 kDA, Merck Millipore, Billerica, MA, USA). Finally, the concentrated solution (*Morchella* EVs) was stored in a refrigerator at – 80 °C for backup.

The morphology of the extracellular vesicles of *Morchella* was observed by TEM (Nippon Electronics Co., Ltd., JEM-2100 PLUS, Akishima City, Tokyo, Japan) using negative staining technique. A small amount of EVs suspension was drawn onto the surface of the copper mesh and left for 10 min, and the excess liquid was blotted out using filter paper. Next, phosphotungstic acid was added dropwise for 10 min, and the excess liquid was blotted out again using filter paper, and then dried and observed using transmission electron microscopy. The extracted extracellular vesicles of *Morchella* were diluted 100-fold and added to the cuvette, and their particle size was measured using a Malvern Zeta potential analyzer (Malvern Zetaszier Nano-ZS, Malvern, UK). BCA kit used for quantitative analysis of *Morchella* EVs.

### Cell culture

RAW264.7 cells are a mouse macrophage cell line derived from the Stem Cell Bank of the Chinese Academy of Sciences (Shanghai, China). The cells were cultured in DMEM medium supplemented with 10% fetal bovine serum and placed in a humidified 5% CO_2_ incubator at 37 °C.

### Cellular uptake studies

RAW264.7 cells were inoculated in 6-well plates and incubated in CO_2_ incubator at 37 ℃ for 24 h. Take the EV suspension in a centrifuge tube, add DIO staining solution on ice without light for 30 min, and then resuspend the precipitate after ultracentrifugation with appropriate amount of PBS to be the stained EVs. Add 5 μg/ml EVS stained with DIO dye, and observe the cell uptake at 0 h, 6 h, 12 h and 24 h respectively. The extracellular vesicle-treated cells were first fixed using 4% Paraformaldehyde Fix Solution, then the nuclei were stained using DAPI dye, and finally determination of extracellular vesicle uptake by the location of fluorescence using two-photon confocal laser microscopy (LSM 880NLO, Carl Zeiss, Oberkochen, Germany).

### Macrophages proliferation assay

The MTT method was used to detect the proliferation of RAW264.7 cell. The cultured RAW264.7 cells were uniformly added to 96-well plates and incubated for 24 h with different concentrations of *Morchella* EVs. After incubation with MTT for 4 h, the crystals were dissolved using DMSO, shaken well and the absorbance values were measured at 570 nm using enzyme-labeled instrument (Bio Tek Epoch2, Vermont, USA) [48, 49].

### Measurement of inflammatory mediators

The cultured RAW264.7 cells were preincubated with different concentrations of *Morchella* EVs for 1 h, and then incubated with LPS stimulation (1 µg/ml) for 24 h. The supernatant was collected and NO content was determined by NO assay kit. The NO content was calculated by comparing with the standard curve [50]. The change of ROS content in the cells was measured by flow cytometry (BD FACSVerse) with ROS detection kit. The experimental procedure was kept as light-protected as possible.

### RT-qPCR analysis

Total RNA was extracted from RAW264.7 macrophages by Trizol method. The quality, purity and concentration of extracted RNA were determined by agarose gel electrophoresis and ultra-micro spectrophotometer. The RNA is reversely transcribed into cDNA, packaged and stored. Using cDNA as template and β-actin as internal reference, RT-qPCR analysis was performed. Finally, the 2^−∆∆CT^ method is used to quantify the obtained data [51]. RT-qPCR primers are as follows:

β- actin- F: 5′-CCATCTACGAGGGCTAT- 3′ β- actin -R: 5′-TCACGCACGATTTCC-3′;

TNF-α- F: 5′- GGCTTCCAGAACTCC- 3′ TNF-α- R: 5′- CAGGCTTGTCACTCG-3′;

iNOS- F: 5′- GGACGAGACGGATAG- 3′ iNOS- R: 5′- GGCTTCAAGATAGGGA-3′;

IL-6—F: 5′- CACTCCCAACAGACC-3′ IL-6 -R: 5′- CTCATTTCCACGATTT-3′;

COX-2- F: 5′- GATTGACAGTCCACCTA-3′ COX-2- R: 5′- GCTCCTTATTTCCCT-3′.

### Western blot

The concentration of extracted protein was measured by BCA method. The protein bands were isolated by SDS-PAGE gel electrophoresis and transferred into PVDF membrane (0.45 µm). At room temperature, the PVDF membrane containing the target protein was immersed in the Blotting-Grade blocker (BIO-RAD, California, USA) for 2 h. After blocking, wash three times with buffer solution (TBST). At 4 ℃, the specific primary antibody was incubated overnight. They were washed with buffer solution (TBST) and then incubated with the second antibody for 4 h at room temperature. CD81, CD63 and Phospho-p38 MAPK antibody are rabbit-derived polyclonal antibody purchased from Affinity Biosciences. p38 MAPK antibody, MK2 antibody and β-actin antibody are mouse-derived monoclonal antibodies purchased from Proteintech Group. Secondary antibodies are goat anti-rabbit HRP (Affinity Biosciences) and goat anti-mouse HRP (Proteintech Group), respectively. After washing with buffer solution for three times, test results were obtained by gel imaging system (BIO-RAD ChemiDoc XRS +). Images and results were quantified with Image J software.

### Statistical analysis

The statistical analysis was performed using GraphPad Prism software version 5.0.1 (GraphPad Software, San Diego, CA). The experiments were carried out in triplicate (3 independent experiments), and the graphs present means ± SD (standard deviations).

## Results

### Isolation and characterization of *Morchella* EVs

**Fig. 1:**
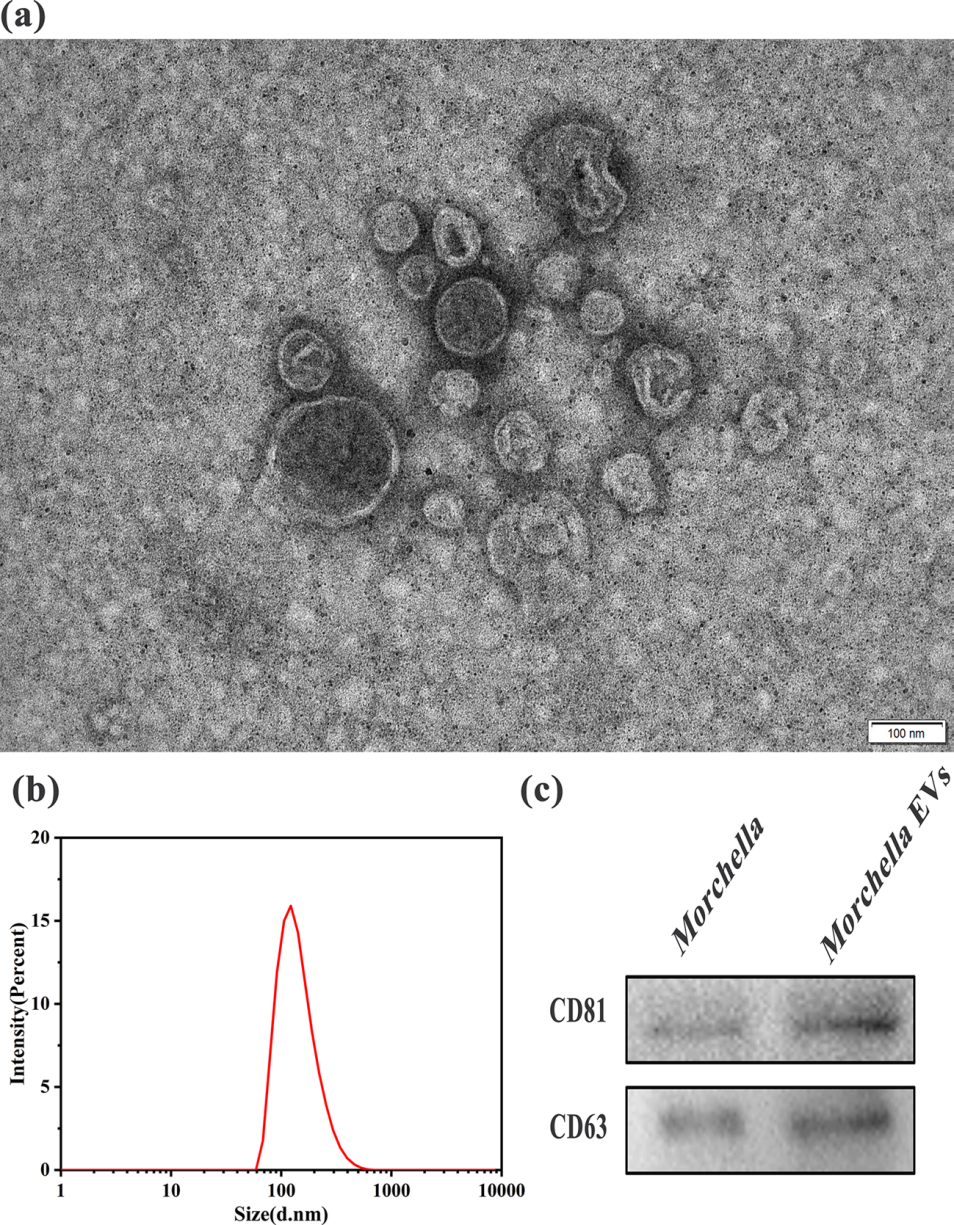
Characterization of extracellular vesicles of *Morchella* isolated from the supernatant of PDA liquid medium. **a** Characterization of TEM topography. **b** Characterization of particle size. **c** Characterization of EVs marker proteins

To confirm whether *Morchella* is able to produce extracellular vesicles, conidium of *Morchella* were cultured. Ultracentrifugation of the culture supernatant to obtain the extracellular vesicles of *Morchella*. *Morchella* EVs were purified by using size-based column chromatography. After negatively staining it, use TEM to observe its morphology and it can be seen that there are round-shaped small vesicles with a bilayer membrane structure, and the inside of the small vesicle structure has different electron microscope densities. (Fig. 1a) The particle size of purified extracellular vesicles was determined by dynamic light scattering method. The results showed that the particle size of *Morchella* extracellular vesicles is mainly distributed between 70 and 400 nm, and the majority of the vesicles were about 120 nm in size. (Fig. 1b) We examined the expression of CD81 and CD63, in *Morchella* as well as in EVs by the Western Blot method. The experimental results are shown in Fig. 1c: CD81 and CD63 proteins were expressed in both EVs and *Morchella*, and both proteins were higher expressed in EVs. The above experimental results show that *Morchella* can produce extracellular vesicles and the extracted extracellular vesicles of *Morchella* were tested by a BCA kit and the concentration was 0.28 μg/μl.

### Intracellular imaging of *Morchella* EVs

The internalization performances of *Morchella* EVs into RAW264.7 cells were studied using a two-photon confocal laser microscope. Dio dye stain *Morchella* EVs membrane and then co-incubated with RAW264.7 cells. The nuclei of RAW264.7 cells were stained with DAPI. Supplementary Fig. S1 proves that *Morchella* EVs can enter RAW264.7 cells and accumulate in the cells.

### Effect of *Morchella* EVs on RAW264.7 cells viability

The potential cytotoxicity of RAW264.7 cells treated with different concentrations of EVs were observed by MTT assay after 24 h. The results showed that the proliferation of RAW264.7 could not be inhibited when the concentration of *Morchella* EVs was less than 3.5 μg/ml. However, at 7 and 14 μg/ml, the cell viability decreased to 80%, which had a significant effect on cell viability (Supplementary Fig. S2). Therefore, 3.5 μg/ml *Morchella* EVs will be used as the maximum concentration of the experimental group in subsequent experiments. We examined the cell survival rate of RAW264.7 cells after 48 h of *Morchella* EVs treatment in the course of conducting the experiment. As shown in Supplementary Fig. S3 when the concentration of *Morchella* EVs was small, it had no effect on the survival rate of RAW264.7 cells. When the concentration was higher than 7 μg/ml, the cell survival rate decreased to less than 80%. The survival rate of RAW264.7 cells treated with *Morchella* EVs for 24 h was not significantly different compared to treatment for 48 h, so our treatment time was set at 24 h. The percentage of complete cell survival is shown in Table S1. Our results showed that the survival rate of RAW264.7 cells was significantly decreased under high concentration of *Morchella* EVs, while low concentration of *Morchella* EV had no effect on the survival rate of RAW264.7 cells.

### Effects of *Morchella* EVs on NO and ROS production

**Fig. 2:**
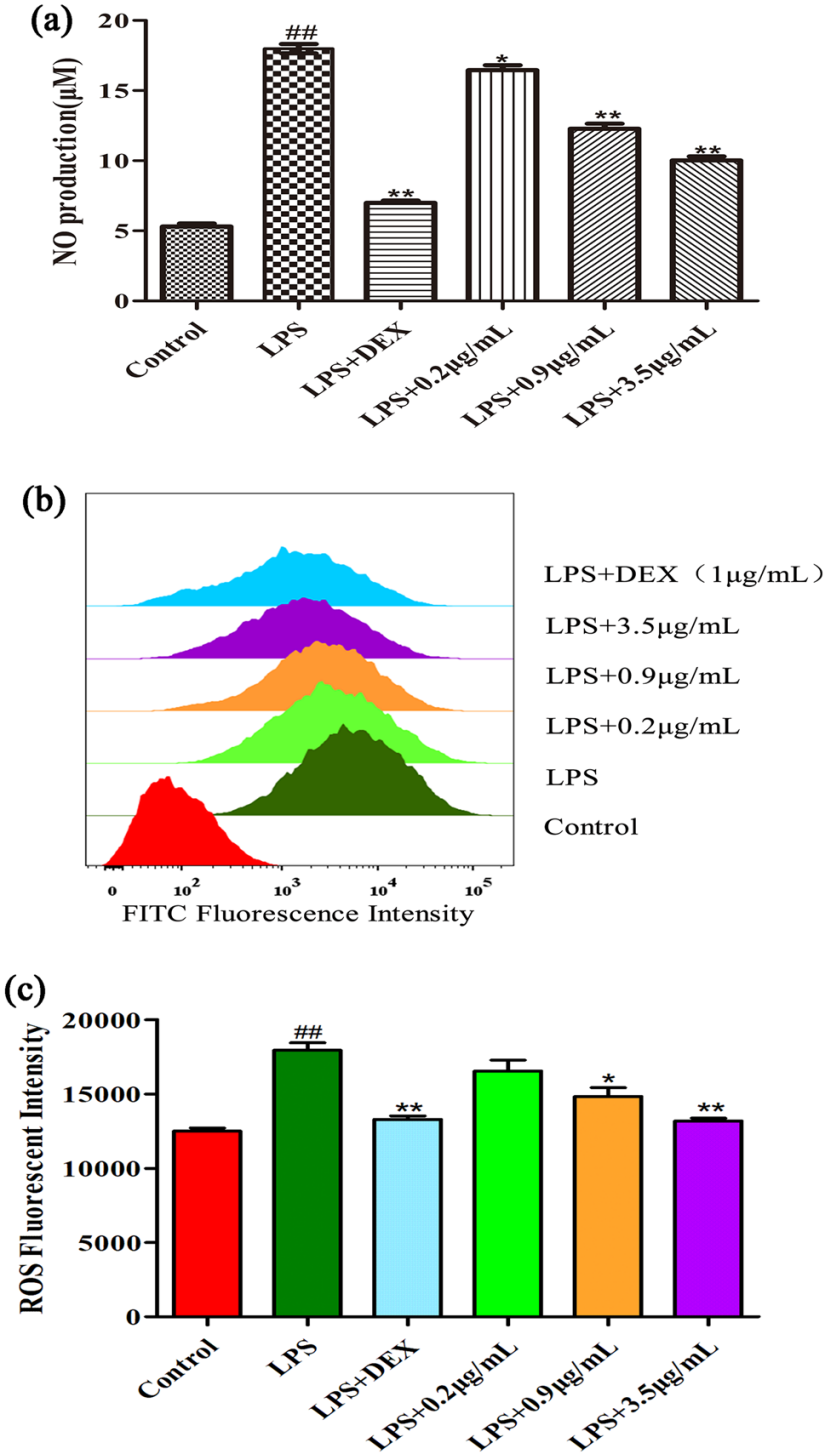
Effect of *Morchella* EVs on NO and ROS production in LPS-stimulated RAW264.7 cells. **a** NO production in LPS-induced RAW264.7 cells treated with different concentrations of *Morchella* EVs. **b**, **c** Flow cytometry detection of ROS production. The results were expressed as means ± SD (*n* = 3), (*p < 0.05 and ** *p* < 0.01 vs. LPS group, and ^##^ *p* < 0.01 vs. control group)

First, we simulated a chronic inflammatory microenvironment by inducing RAW264.7 macrophages with LPS. Under this condition, we explored the effect of EVs on NO and ROS production by RAW264.7 macrophages. Because dexamethasone is closely associated with the treatment of inflammation-related diseases, we used it as a positive control. It was found (as shown in Fig. 2a) that NO release from RAW264.7 macrophages of the LPS group was significantly repressed in a concentration-dependent manner after pretreatment with *Morchella* EVs. At the same time, DCF-DA fluorescence detection was performed on LPS-induced RAW264.7 macrophages by flow cytometry (BD FACSVerse), which reflect the production of intracellular ROS. The results showed that the ROS content in RAW264.7 cells was significantly higher in the LPS group compared with the control group, while the EVs of *Morchella* in the experimental group could significantly inhibit LPS-induced ROS production in a dose-dependent manner. (Fig. 2b, c) In conclusion, *Morchella* EVs inhibited NO and ROS production of LPS-induced Raw267.4 cell in a dose-dependent manner.

### Anti-inflammatory effect of *Morchella* EVs mediated by modulation of immune-associated gene expression

**Fig. 3:**
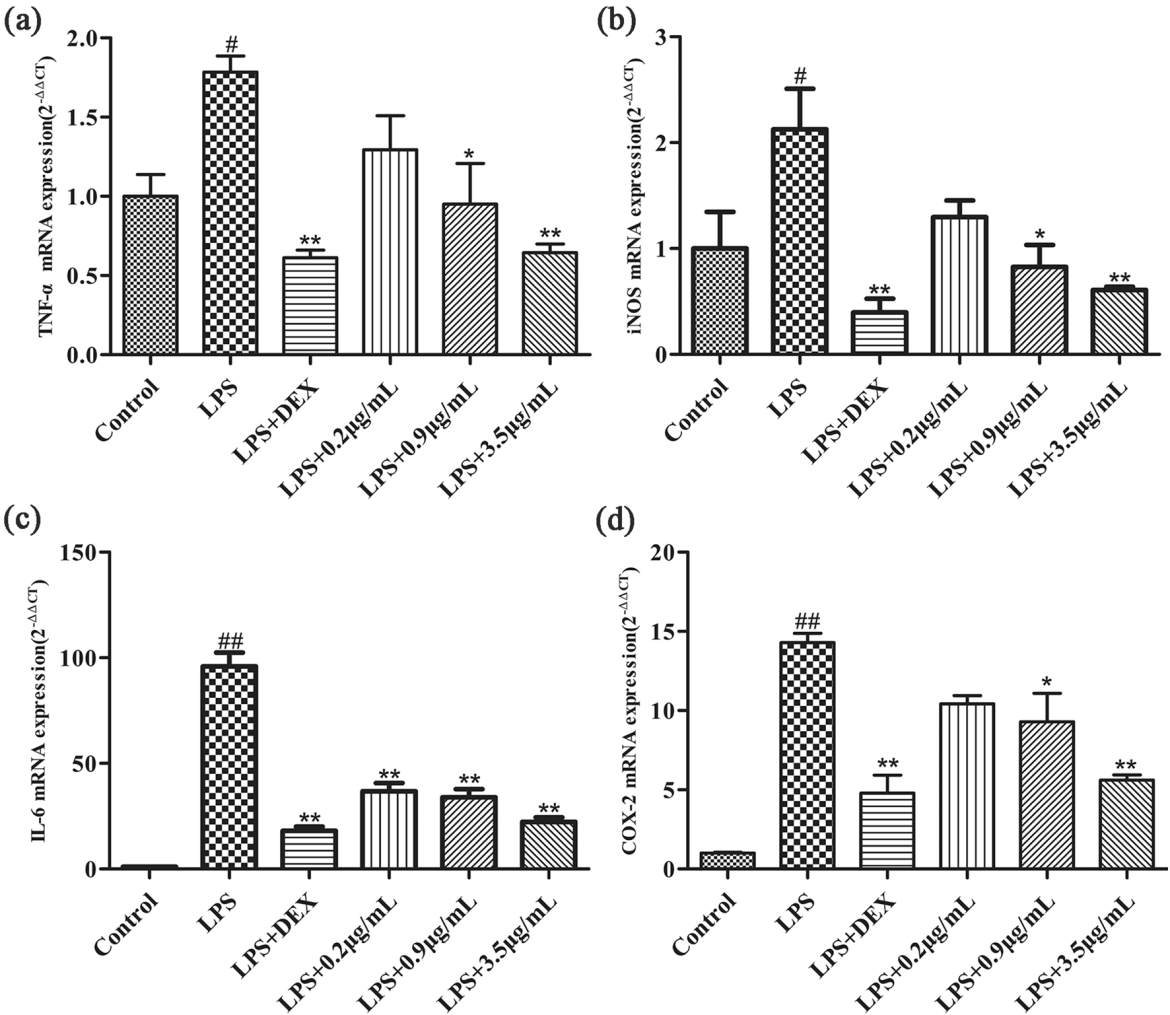
Effects of *Morchella* EVs on the expression levels of immune-associated genes in LPS-stimulated RAW264.7 cells. **a **TNF-α, **b** iNOS, **c** IL-6, **d** COX-2. The results were expressed as means ± SD (*n* = 3), (*p < 0.05 and ** *p* < 0.01 vs. LPS group, and ^##^*p* < 0.01 vs. control group)

The expression of immune-related genes in different treatment groups was detected by real-time quantitative PCR. The results showed that the expression of immune-related genes, including TNF-α, iNOS, COX-2 and IL-6, were significantly up-regulated in RAW264.7 macrophages after LPS treatment. In contrast, RAW264.7 macrophages in the LPS group were treated with *Morchella* EVs and the expression of most tested genes was decreased. The mRNA expression levels of intracellular inflammatory mediators and pro-inflammatory cytokines were significantly reduced in a concentration-dependent manner after treatment with extracellular vesicles of *Morchella*. In summary these results clearly show that *Morchella* EVs can inhibit inflammation via inhibiting the expression of genes related to inflammatory response. As shown in (Fig. 3a, b, c &d).

### Anti-inflammatory effects of *Morchella* EV involve the p38 MAPK signaling pathways

**Fig. 4:**
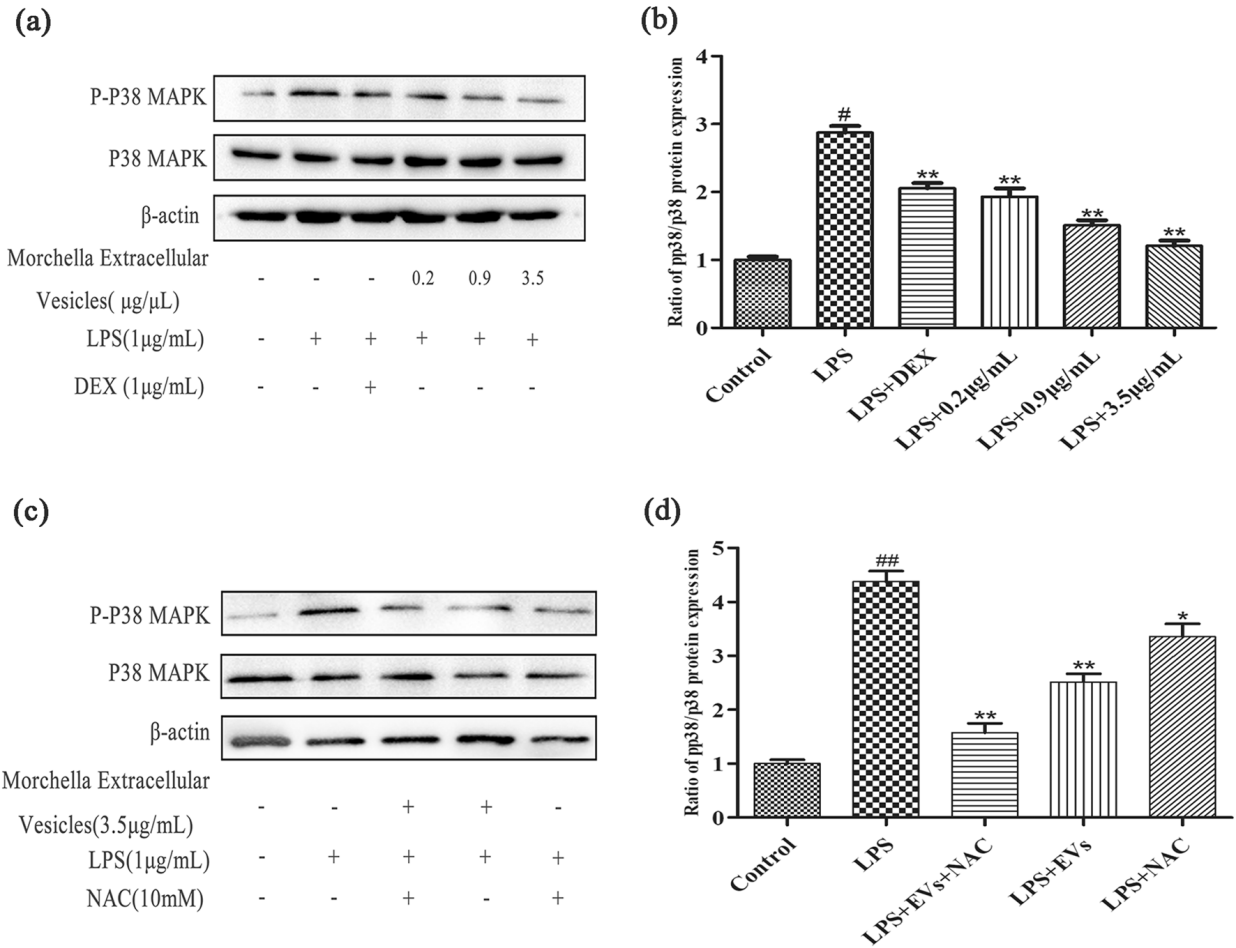
**a**, **b** Effect of *Morchella* EVs on the protein levels in the p38 MAPK pathways in LPS-stimulated RAW264.7 cells as determined by western blotting. **c**, **d** Effect of ROS on the protein levels in the p38 MAPK pathways in LPS-stimulated RAW264.7 cells as determined by western blotting. The results were expressed as means ± SD (*n* = 3), (*p < 0.05 and ** *p* < 0.01 vs. LPS group, and ^##^*p* < 0.01 vs. control group)

For the anti-inflammatory mechanism of the extracellular vesicles of *Morchella* involved in the relevant signaling pathways, we used Western blotting to detect. The results showed that the phosphorylation level of p38 protein was significantly reduced in LPS-induced RAW264.7 cells after treatment with *Morchella* EVs. Compared with the positive control group, *Morchella* EVs decreased the phosphorylation level of p38 in a concentraion dependent manner. (Fig. 4a, b) Therefore, we speculated that the p38 signaling pathway is a major pathway in the anti-inflammatory process of *Morchella* EVs.

So as to verify whether the regulation of the p38 MAPK signaling pathway is related to EVs regulating the generation of ROS, the ROS inhibitor NAC was used to inhibit the ROS produced by LPS-induced cells [52]. The experimental results as shown in Fig. 4c, d show that the p38 MAPK signaling pathway was inhibited when NAC was added, indicating that the reduction in ROS levels also inhibits the p38 MAPK signaling pathway. Compared with the addition of NAC and EVs alone, the p38 MAPK signaling pathway was inhibited more strongly when both NAC and EVs were added, which indicates that NAC and EVs can play a synergistic role in inhibiting the p38 MAPK signaling pathway. In short, *Morchella* EVs achieved anti-inflammatory effects by directly inhibiting p38 MAPK signaling pathway and indirectly regulating p38 MAPK signaling pathway by inhibiting ROS production in LPS-induced RAW264.7 cells.

MAPK-activated protein kinase 2 (MK2) is an important downstream substrate of the p38 MAPK signaling pathway [53, 54]. MK2 is known to be involved in many cellular processes including stress and inflammatory responses, nuclear export, gene expression regulation, and cell proliferation [55–57]. To further prove our results, we examined the expression of MK2, a protein downstream of the p38 MAPK signaling pathway. The experimental results as shown in Fig. S3 show that MK2 protein expression was inhibited when NAC was added, and the MK2 protein expression was more strongly inhibited when both NAC and EVs were added, which indicates that NAC and EVs can play a synergistic role in inhibiting MK2 protein expression. It further demonstrates that *Morchella* EVs achieved anti-inflammatory effects by directly inhibiting p38 MAPK signaling pathway and indirectly regulating p38 MAPK signaling pathway by inhibiting ROS production in LPS-induced RAW264.7 cells.

## Discussion

*Morchella* is rich in nutrition and contains dietary fiber, vitamins, minerals and essential amino acids and has high medicinal value [58]. Previous studies have shown that *Morchella* polysaccharides act as immune adjuvants by enhancing the immune response [59]. Extracellular vesicles contain a variety of molecules, which may contribute to changes in the host immune system [60, 61]. This article introduces the preparation and isolation of *Morchella* EVs, Characterized by TEM and dynamic light scattering method, which proves that *Morchella* can produce extracellular vesicles. This is the first study to demonstrate that extracellular vesicles can be isolated from *Morchella*.

In this study, we simulated a chronic inflammatory microenvironment by inducing RAW264.7 macrophages with LPS. Under this environment, we explored the anti-inflammatory effect and regulatory mechanism of *Morchella* EVs on RAW264.7 macrophages. Because dexamethasone is closely associated with the treatment of inflammation-related diseases, we used it as a positive control. In addition, NO and ROS participate in the immune system regulation process. NO, a cellular messenger molecule, involves in the regulation of immune system [62]. ROS can be used as an initial signal to activate the non-specific immune system and play a key role in physiological activities such as tissue damage repair by acting as an initial signal to activate the non-specific immune system [63]. The results showed that *Morchella* EVs significantly decreased NO and ROS production and the expression of immune-associated genes in LPS-stimulated Raw264.7 cells. It indicated that *Morchella* EVs can be used as a potential anti-inflammatory regulators.

Previous studies have shown that MAPKs signaling pathway is a participant in inflammation [64]. The activation of MAPKs signaling pathway is a marker of LPS-induced macrophage signal transduction [65]. MAPKs is a protein family of highly conserved serine/threonine kinases, including subgroups p38, ERK1/2, and JNK [66]. The p38 MAPK pathway mainly transduces inflammatory cytokines and multiple types of cell stress signals, and many studies have proven that blocking the p38 cascade can reduce inflammatory response [65, 67]. We detects the expression of p38 protein and pp38 protein through Western Blot. The results showed that the phosphorylation level of p38 protein was significantly reduced in LPS-induced RAW264.7 cells after treatment with *Morchella* EVs. Compared with the positive control group, *Morchella* EVs decreased the phosphorylation level of p38 in a concentraion dependent manner (Fig. 4a, b). Therefore, we speculated that the p38 signaling pathway is a major pathway in the anti-inflammatory process of *Morchella* EVs.

Studies have found that LPS stimulates macrophages to promote the production of ROS, and ROS can also mediate the activation of relevant signal pathways and transcription factors as a second messenger, thus regulating the occurrence and development of inflammatory responses [27, 68, 69]. We suspect that the inhibitory effect of EVs on the p38 MAPK signaling pathway is related to the regulation of ROS production by EVs? Therefore, we use ROS inhibitor NAC(10 mM) to inhibit the ROS produced by LPS-induced cells [52, 70]. Compared with the addition of NAC and EVs alone, the p38 MAPK signaling pathway was inhibited more strongly when both NAC and EVs were added, which indicates that NAC and EVs can play a synergistic role in inhibiting the p38 MAPK signaling pathway (Fig. 4c, d). To further prove our results, we examined the expression of MK2, a protein downstream of the p38 MAPK signaling pathway and the results showed that MK2 protein was also inhibited. In a word, *Morchella* EVs can inhibit LPS-induced inflammatory response in two ways. One way is to suppress inflammation by inhibiting ROS production, the other way is by regulating the p38 MAPK signaling pathway.

## Conclusion

Our study demonstrated that *Morchella* EVs achieved anti-inflammatory effect by preventing the p38 MAPK signaling pathway and production of ROS in RAW264.7 macrophages. Our current findings suggest that *Morchella* EVs can be used as a potential anti-inflammatory substance in the treatment of inflammatory diseases. However, further studies are needed regarding the potential benefits of extracellular vesicles of *Morchella* in primary macrophages and in vivo studies, and which biomolecules inside the vesicles are exactly involved in anti-inflammation.

## Supplementary Information

Below is the link to the electronic supplementary material.

Supplementary file1 (DOCX 1878 kb)

## Data Availability

All the data are contained in the manuscript file and will be made available from the corresponding author upon reasonable request.
