# Supplementary material for: Anti-inflammatory effects of extracellular vesicles from Morchella on LPS-stimulated RAW264.7 cells via the ROS-mediated p38 MAPK signaling pathway
Source: Mol Cell Biochem. 2022 Jul 7;478(2):317–27. doi: 10.1007/s11010-022-04508-y (PMC9886593; doi:10.1007/s11010-022-04508-y)
Supplement: Supplementary file 1 — Supplementary file1 (DOCX 1878 kb) [file 11010_2022_4508_MOESM1_ESM.docx]

**Molecular and Cellular Biochemistry**

**Anti-Inflammatory Effects of Extracellular Vesicles from *Morchella* on LPS-Stimulated RAW264.7 Cells via the ROS-mediated p38 MAPK signaling pathway**

Qi Chen^#^, Chengchuan Che^#^, Shanshan Yang, Pingping Ding, Meiru Si and Ge Yang*

College of Life Sciences, Qufu Normal University, Qufu 273165, Shandong, People’s Republic of China

#These authors contributed equally to this work and should be considered co-first authors.

*Corresponding author:

College of Life Sciences, Qufu Normal University, Qufu 273165, Shandong, People’s Republic of China; E-mail addresses: yangge@qfnu.edu.cn

**Supplementary Fig. S1**

**
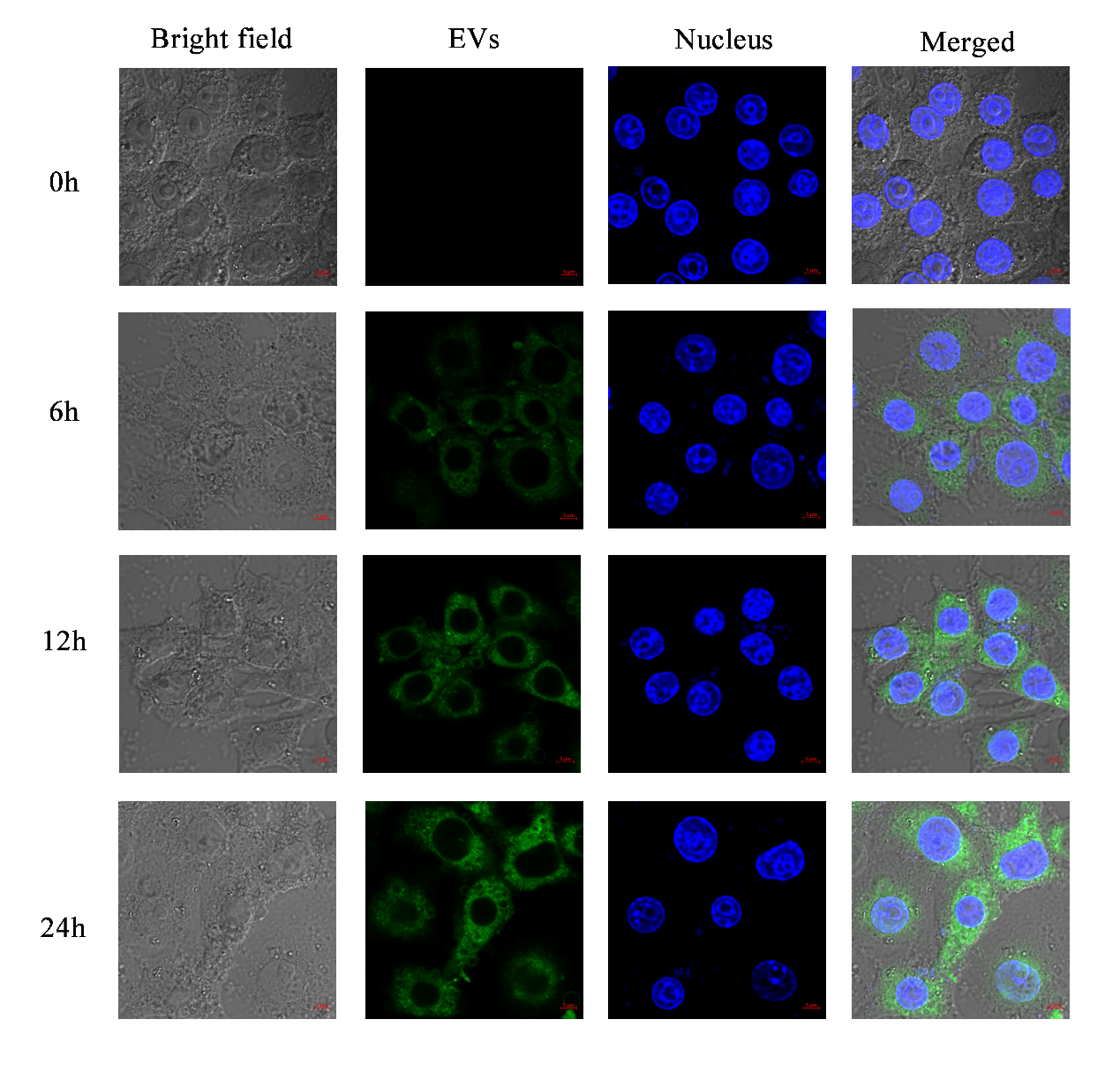
**

**Supplementary Fig. S1** The distribution of *Morchella* extracellular vesicles in RAW264.7 cells incubated at 0h, 6h, 12h and 24h.

**Supplementary Fig. S2**


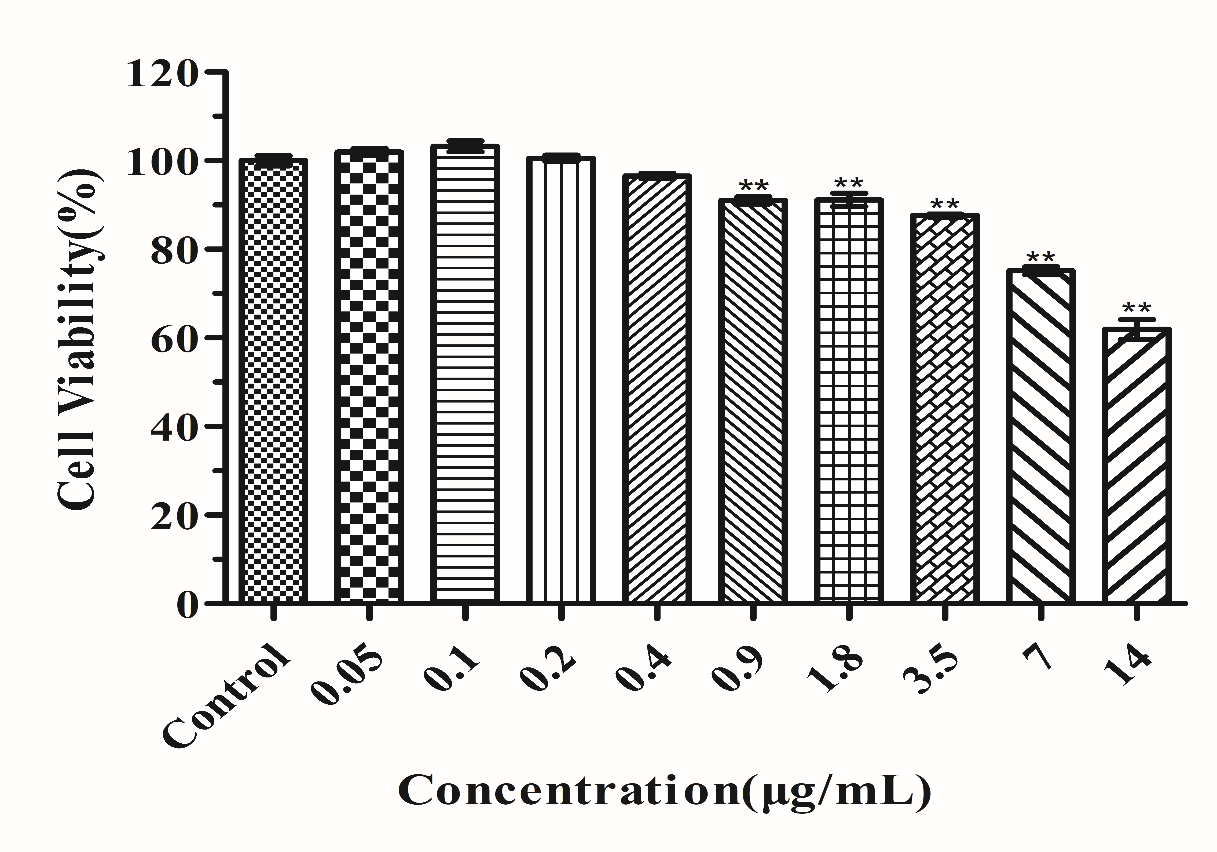


**Supplementary Fig. S2** The MTT method was used to detect the effect of different concentrations of *Morchella* extracellular vesicles treatment for 24 hours on the viability of RAW264.7 cells. The results are expressed as mean ± standard deviation (n=3), **p<0.01, which is extremely significant compared with the control group.

**Supplementary Fig. S3**

**
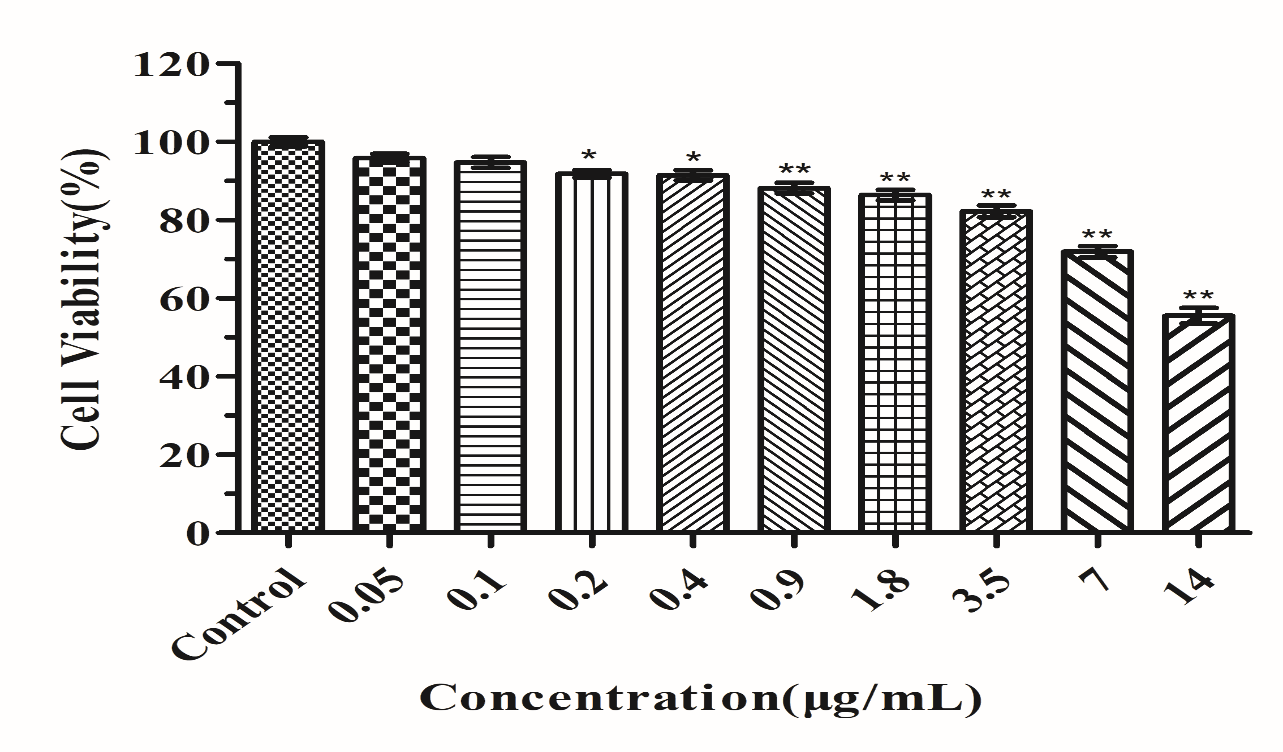
**

**Supplementary Fig. S3** The MTT method was used to detect the effect of different concentrations of *Morchella* extracellular vesicles treatment for 48 hours on the viability of RAW264.7 cells. The results are expressed as mean ± standard deviation (n=3), **p<0.01, which is extremely significant compared with the control group.

**Supplementary Fig. S4**


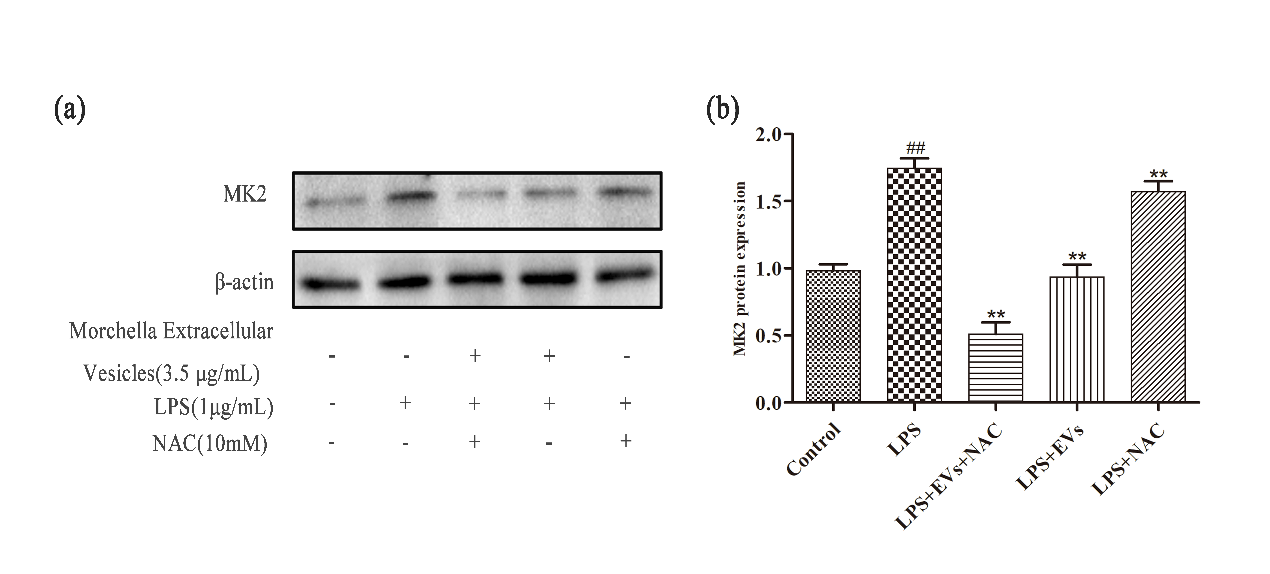


**Supplementary Fig. S4** Effect of *Morchella* EVs on the protein levels of MK2 in LPS-stimulated RAW264.7 cells as determined by western blotting. The results were expressed as means ± SD (n = 3), (*p< 0.05 and ** p < 0.01 vs. LPS group, and ^##^p < 0.01 vs. control group)

**Supplementary Table. S1**

| *Morchella* EVs Concentration (μg/mL) | RAW264.7 cell survival rate (%) at 24 h |
| --- | --- |
| 0 | 100 |
| 0.05 | 101.97±2.18 |
| 0.1 | 103.25±3.42 |
| 0.2 | 100.55±1.88 |
| 0.4 | 96.58±1.67 |
| 0.9 | 91.05±2.15 |
| 1.8 | 91.17±4.19 |
| 3.5 | 87.63±1.05 |
| 7 | 75.16±2.60 |
| 14 | 61.93±6.37 |

| *Morchella* EVs Concentration (μg/mL) | RAW264.7 cell survival rate (%) at 48h |
| --- | --- |
| 0 | 100 |
| 0.05 | 95.84±3.16 |
| 0.1 | 94.75±3.95 |
| 0.2 | 91.80±2.80 |
| 0.4 | 91.45±3.78 |
| 0.9 | 88.18±3.81 |
| 1.8 | 86.42±3.89 |
| 3.5 | 82.25±4.13 |
| 7 | 71.91±4.13 |
| 14 | 55.55±5.58 |

**Supplementary Table. S1** Cell viability of *Morchella* EVs treated RAW264.7 cells after 24 and 48 hours.
